# Learning to Detect Cracks on Damaged Concrete Surfaces Using Two-Branched Convolutional Neural Network

**DOI:** 10.3390/s19214796

**Published:** 2019-11-04

**Authors:** Jieun Lee, Hee-Sun Kim, Nayoung Kim, Eun-Mi Ryu, Je-Won Kang

**Affiliations:** 1 Department of Electrical and Electronic Engineering, Ewha Womans University, Seoul 03760, Korea; leeje2993@gmail.com (J.L.); 12skdud21@naver.com (N.K.); 2 Department of Architectural and Urban Systems Engineering, Ewha Womans University, Seoul 03760, Korea; heesun531@gmail.com (H.-S.K.); gogo5423@nate.com (E.-M.R.)

**Keywords:** deep learning, crack detection, convolutional neural network, edge detection, fire-damaged concrete, image processing

## Abstract

Image sensors are widely used for detecting cracks on concrete surfaces to help proactive and timely management of concrete structures. However, it is a challenging task to reliably detect cracks on damaged surfaces in the real world due to noise and undesired artifacts. In this paper, we propose an autonomous crack detection algorithm based on convolutional neural network (CNN) to solve the problem. To this aim, the proposed algorithm uses a two-branched CNN architecture, consisting of sub-networks named a crack-component-aware (CCA) network and a crack-region-aware (CRA) network. The CCA network is to learn gradient component regarding cracks, and the CRA network is to learn a region-of-interest by distinguishing critical cracks and noise such as scratches. Specifically, the two sub-networks are built on convolution-deconvolution CNN architectures, but also they are comprised of different functional components to achieve their own goals efficiently. The two sub-networks are trained in an end-to-end to jointly optimize parameters and produce the final output of localizing important cracks. Various crack image samples and learning methods are used for efficiently training the proposed network. In the experimental results, the proposed algorithm provides better performance in the crack detection than the conventional algorithms.

## 1. Introduction

Crack information can help timely and proactive management of concrete structures, and image sensors are economically useful to detect the cracks on concrete surface as compared to other sensors [1,2,3,4]. However, the conventional process of the visual inspection is too time-consuming since it needs manual tracing of cracks on the surface image. Crack detection algorithms can perform quantitative analysis on the strengths or lengths of edges to estimate a degree of safety. In practice, such autonomous crack assessment is effectively used for safety diagnosis of concrete structures such as bridges [1], nuclear plants [2], pavements [3], and tunnels [4] through image sensors.

**Figure 1:**
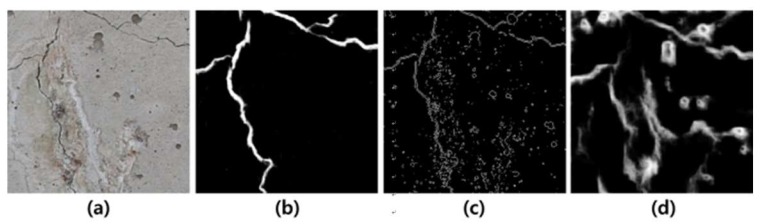
(**a**) The original concrete surface, (**b**) the crack map manually traced by an expert, (**c**) edge detection by Sobel mask, and (**d**) by Holistic Edge Detection (HED) [9].

The main challenge in crack detection is to identify only the important cracks whose widths and lengths are greater than some thresholds, specified by a safety instruction [5]. Earlier crack detection algorithms use edge detection and morphological image processing algorithms such as Canny detector, Sobel mask, and Laplacian of Gaussian (LoG) [6,7,8]. However, many noises or other tiny pores and scratches on the surfaces make cracks difficult to be detected in the real world. The task is even more challenging when the surfaces of concretes are damaged by various factors [9,10,11,12,13,14]. For instance, Figure 1 shows parts of fire-damaged concretes. The detection accuracy is significantly degraded by combustion as compared to the cracks pointed by domain experts. Many image processing algorithms are actually sensitive to such noise and undesired artifacts by damages.

Recently, Convolutional Neural Network (CNN) has been actively applied to various image processing and understanding problems such as edge detection [9], saliency detection [10], semantic segmentation and recognition [11]. The CNN uses automatic hierarchical feature learning in an end-to-end manner to allow for understanding different contexts in an image. Holistic Edge Detection (HED) [9] develops a CNN-based edge detection system, combining multi-scale and multi-level visual responses in convolution layers. Deep Contour-Aware Network (DCAN) [11] proposes to use multi-level contextual features to accurately detect contours and separate clustered objects. In [10], Pixel-wise Contextual Attention Network (PiCANet) is proposed to detect important or salient regions in an image.

In this paper, we propose an autonomous and reliable crack detection algorithm using CNN, extended from the preliminary work [12]. Even though a domain expert can easily identify critical cracks that can have significant impact on the safety evaluation of a concrete surface, it is a much more difficult task for an autonomous system due to undesired artifacts on the damaged concrete surfaces [15,16,17]. To solve the problem, the proposed algorithm is designed for localizing important cracks based on the recent advances in deep learning research. Our previous work focuses on safety evaluation of fire-damaged concretes by showing the correlation between the lengths of cracks, durations, and temperatures and structural performance. However, in this work, we rather show more thorough ideas on autonomous crack detection using deep learning. Experimental results conducted with various crack datasets show the proposed algorithm provides more accurate and reliable performance in crack detection compared to previous works.

Our contribution in this paper is as follows. We use a two-branched CNN architecture to efficiently distinguish the relevant crack and the other components such as noise and edge-like image components on the concrete surfaces. The intuition behind the proposed model is to use noise-suppression and region detection, inspired by old wisdom on conventional edge detection methods and multi-channel network architecture [18,19]. Specifically, a branch of the proposed network is to detect edge or contours that are considered as the most prominent components in cracks, and the other branch is to identify a region-of-interest as in semantic segmentation. The features learned from the two different networks are combined for identifying the important cracks. Data acquisition and training strategy are important to overcome an over-fitting problem in deep learning and appropriately validate the performance. Therefore, to facilitate learning, we conduct fire experiments for ourselves to obtain more crack image samples in the real world. It is noted that the fire-damaged concretes show many detailed cracks with combustion, so the reliable crack detection algorithm is mattered. Furthermore, the proposed algorithm uses skip connections made with convolution and deconvolution operations that can transfer the crack features trained in the lower layers to the higher layers on top of the U-net architecture [20].

The rest of the paper is organized as follows. In Section 2, we review the previous studies. In Section 3, we explain the proposed method. In Section 4, we show the proposed training strategy and data acquisition. Experimental results are shown in Section 5. We conclude with remarks in Section 6.

## 2. Related Works

### 2.1. Previous Studies in Crack Detection

Earlier crack detection algorithms use edge detection and morphological image processing algorithms. Youm et al. develop a crack detection algorithm, comparing the differences of intensities in neighboring pixels to the pre-defined thresholds to determine a region-of-interest [7]. Similarly, the crack regions are separated with fuzzy c-means clustering and segmentation algorithms [6]. Conventional edge detection algorithms such as canny detector, Sobel mask, and Laplacian of Gaussian (LoG) weighted Haar-like features, and the Hessian matrix-based operations are used [8,21]. They offer post-processing such as delate operator because the detection algorithms using pixel intensities and gradients are often vulnerable to luminance change, low-contrast images, and complex backgrounds. Cho et al. propose an edge-based crack detection method using crack width transform [22]. Liang et al. propose crack extraction and identification method based on machine vision [23].

Machine learning has been widely used for the crack detection, consisting of two steps, i.e., feature extraction and decision. Various hand-crafted features considering edge components of the cracks are proposed. Li et al. use a local binary pattern (LBP) to express key attributes of lengths and widths of the cracks [24]. Zalama et al. use visual features based on Gabor filters for describing anisotropic components of cracks [25]. The feature vectors are trained with support vector machine and neural network to determine the regions of cracks. 

There have been several crack detection studies using CNN. The CNN automatically extracts the features from the raw images regardless of professional knowledge on a target structure. In [3,4], the CNN-based crack detectors can perform the safety diagnosis even though geometry information of target concrete structures is unknown. In [12], damaged areas in concrete structures are localized to evaluate a degree of safety. In [2], a CNN-based crack detection algorithm and a fusion method using naive Bayesian algorithm are proposed to identify crack components in nuclear power plants. In [13] a deep learning-based segmentation algorithm is proposed to identify cracks in a tunnel. In [3,14,15,16,17] the CNN has been used for crack detection by the supervision of block-based classification. The works classify image blocks of concrete surfaces into crack or non-crack regions using the softmax layer. The networks produce the predicted scores, which are represented as pixel intensities between 0 and 255, e.g., a brighter pixel is more likely to be a crack region [26]. These tasks can be achieved with minimizing cross-entropy loss in the training [27]. The classification performance of the CNN increases more as the layers are deeper [16]. Accordingly, efficient training methods such as pre-training, transfer learning, batch normalization [28], and drop-out [29] are applied to increase the depths in concrete images [17]. However, such classification approaches may determine the entire region of a block to cracks even though some parts of the blocks are a non-crack region. The classification can be conducted with various sizes of block patches to identify more accurate crack regions. Though the learned features are more robust to noise in the real world data, the previous works have not carefully considered attributes of cracks in the detection. 

### 2.2. Convolution and Deconvolution Architecture in CNN

The CNN has cascaded convolution layers and pooling layers, ending with fully connected layers. The neuron units in convolution layers are connected to local patches consisting of feature maps and convolve the neurons in the previous layers. The convolution and deconvolution architecture, which is an advanced CNN structure developed for various image processing techniques [9,20,30,31] has the symmetric structures of convolution and deconvolution layers in both ends. In theory, the extracted features from the convolution layers play roles in summarizing the inputs into low-dimensional vectors [32]. The vectors can be reconstructed by deconvolution layers to restore the original size.

**Figure 2:**
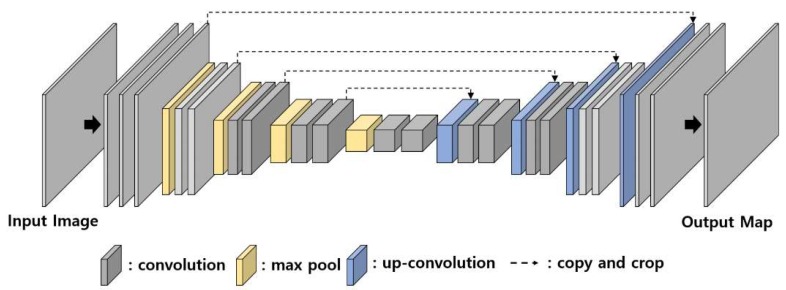
U-net structure [20].

U-net is one of the representative convolution and deconvolution architectures originally proposed for the segmentation task of a biomedical image [20]. U-net has a symmetrical u-shaped structure of convolution layers and deconvolution layers as shown in Figure 2. There are variant forms of the U-net, which are applied to edge detection, segmentation, and saliency detection. Holistic Edge Detection (HED) [9] uses various multi-scale U-net architectures to detect fine edges. The U-net is applied to the segmentation and saliency map detection to identify a perceptually important region in an image [10].

It is emphasized that our work differs from previous works. Our work is different to the recent CNN-based edge or contour detection algorithms [9,11,20] as the proposed technique has noise-suppression or region detection networks to localize only the critical cracks. Furthermore, our work is distinguished from the recent crack detection algorithms using deep learning [2,3,14,15,16,17,33] as it generates a pixel-wise crack map from the combination of two different sub-networks, i.e., one for detecting the crack components and the other for detecting the crack regions. The previous works focus on supervised learning for a classification problem in crack detection.

## 3. Proposed Crack Detection Network

### 3.1. Motivation

**Figure 3:**
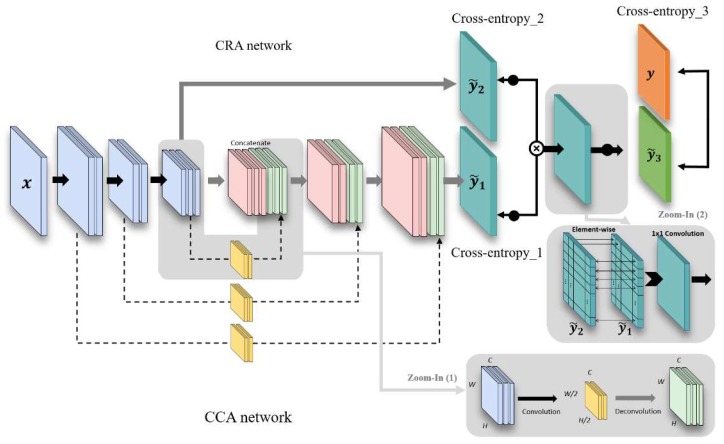
Proposed two stream CNN architecture.

For an efficient crack detection, a trade-off between noise suppression and localization needs to be considered. In other words, the detector may be able to find the precise location of the crack, but the effects of noise increase and vice versa. The problem is challenging especially in crack images as they are captured in the wild and suffer from noise. To solve the problem, we propose a two-branched network architecture as shown in Figure 3, consisting of sub-networks named a crack-component-aware (CCA) network and a crack-region-aware (CRA) network. In one hand, the CCA network is to find a low-level image feature regarding the crack, e.g., the representation of the anisotropy property of the crack as the crack has many edge-like image features. On the other hand, the CRA network is to approximate the region-of-interests by distinguishing crack and non-crack regions. In the CRA network, a higher weight is assigned to a region closer to the crack and vice versa. By combining the outputs of the two sub-networks, the proposed network can suppress small noise in non-crack regions that have been detected in the CCA network to improve the accuracy. 

### 3.2. Architecture Description

#### 3.2.1. Crack-Component-Aware Network

The CCA network consists of seven convolution layers and three deconvolution layers as shown in Table 1. We connect the convolution layer to the deconvolution layer to effectively deliver the trained features in the lower layers to the higher layers, motivated by the symmetric U-net structure [20] and the skip connection. The skip connection is known to improve overall performance with slight increments of computational complexity [19]. 

**Figure 4:**
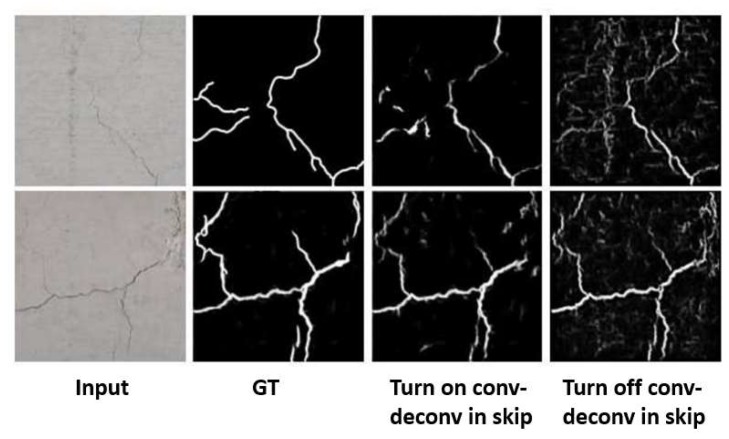
Ablation tests turning on and off the convolution and deconvolution in skip connections.

Motivated by the work in our algorithm, the architecture is designed to combine the output feature map of a convolution layer with that of the corresponding deconvolution layer at the symmetric position. The skip connection is made using internal convolution and deconvolution operations in our algorithm. To be specific, as zoomed in Figure 3, we apply a convolutions layer using kernel size 2 and stride 2 for the internal convolution operation in the skip connection, so we have the half size of feature map as $C_{f}\times \frac{W_{f}}{2}\times \frac{H_{f}}{2}$. For the deconvolution operation in the skip connection, we use a deconvolution layer using kernel size 8 and stride 2 to recover the original feature size $C_{f}\times W_{f}\times H_{f}$. Afterwards, they are concatenated with the existing layers in the deconvolution layers of the CCA. By doing so, the contexts trained in the convolution layer cannot be missed and maintain the important characteristics of real cracks. We also show the ablation tests turning on and off the convolution and deconvolution layers of the skip connections in testing procedures. When turning on the operation, the network can focus on the actual cracks. In the opposite case, it shows other noise components when turning off the function, as shown in Figure 4.

The skip connections are located at the convolution layers before pooling layers. The kernel size in the convolution layer is set to 3. After the deconvolution layers, the CCA network predicts a map that localizes the ground truth of a crack map. We also add a max-pooling layer to every 2 or 3 convolution layers to reduce the number of parameters and prevent an overfitting problem. 

#### 3.2.2. Crack-Region-Aware Network

The CRA network has 10 convolution and pooling layers and one deconvolution layer to restore the same size as the size of an input image. The layers before pooling layer are shared with the CCA network. The network maintains the features of the up-sampled image through the 1 × 1 convolution layer, as shown in Table 2. The network also provides a predicted map that approximates a region of a crack. We set the kernel size to 3.

#### 3.2.3. Combination of CCA and CRA

**Figure 5:**
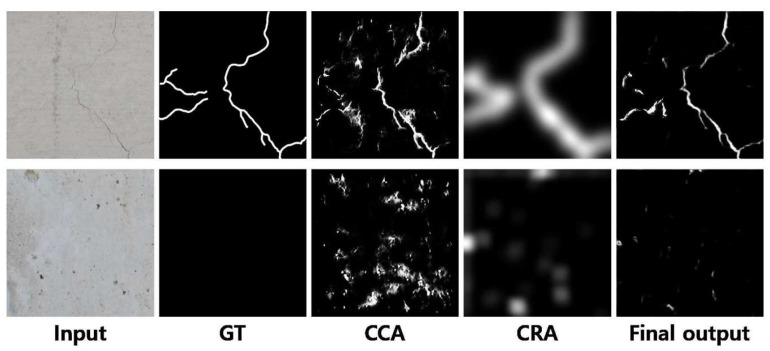
Examples of input images, the ground truth, the CCA output, the CRA output, and the final output (from left to right).

The features trained in CCA and CRA are the same before pooling layer 3 in Table 1 and Table 2, but the feature maps that have passed through the CCA and CRA networks are further computed with element-wise multiplication and additional one 1 × 1 convolution layer to precisely output the map of cracks, as shown in the final output of Figure 5. Figure 5 shows examples of input images and the ground truth and the intermediate outputs of the CCA and the CRA and the final output from the left to the right. The output values of the CRA are adjusted to have the maximum pixel value of 255 in the examples for the purpose of visual comparisons, while they are actually filled with some small values. The features trained in CCA capture detailed edges in concrete surfaces, but the results can include a lot of edges, blobs, and lines. Meanwhile, the CRA cannot extract detailed patterns as shown in the fourth column, while being capable of determining whether the region is important or not. When combining the results, the crack is appropriately extracted in the output. For instance, the input in the second row has none of important cracks, and accordingly, the output map becomes empty even though the CCA points several low-level edge features in the image.

## 4. Training

### 4.1. Crack Data Acquisition

**Figure 6:**
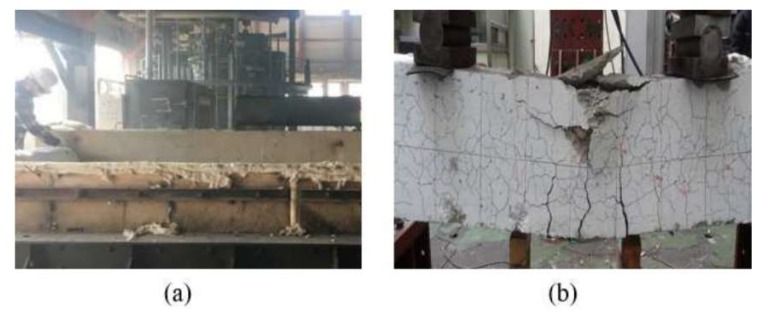
(**a**) Fire experiments to construct the database and (**b**) the concrete structures after the fire.

It is important to use a large number of data samples to train the deep neural network and validate the performance. We show how to acquire crack image samples and manage them for training. We use three crack databases, i.e., Fire Crack Dataset (FCD), CrackForest Dataset (CFD) [34], and AigleRN [35]. The FCD is obtained from fire experiments conducted by the authors to have a sufficient size of datasets. The acquisition process by fire experiments is shown in Figure 6. The CFD and the AigleRN are available online.

#### 4.1.1. Fire Crack Dataset (FCD)

Two concrete structures are made with the height, the width, and the length of 25 cm × 40 cm × 500 cm, and they go through fire experiments to obtain the crack dataset. The fire experiments are conducted for the two concretes around 60 min and 120 min, respectively. The time-temperature curves are controlled to follow ISO-834. High Definition (HD) resolution images of concrete surfaces are obtained from DSLR (Digital Single Lens Reflex) cameras. The camera is a Canon EOS 760D with Canon EF-S 60mm USM Macro lens. The aperture is f2.8. The resolution is 24 megapixels (6000 × 4000 pixels). It is shown in [36] that the detection of fatigue cracks using image processing techniques is feasible in appropriate conditions of camera specification. Our conditions also satisfy the constraints.

**Figure 7:**
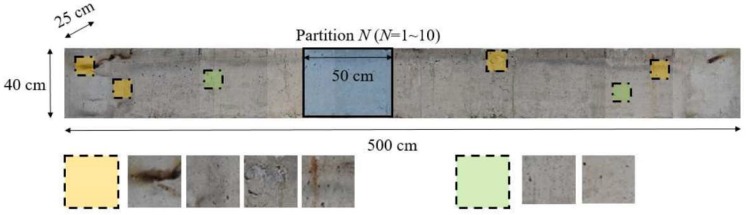
Sample concrete specimen and image acquisition steps.

We take several pictures of the parts of the original concrete specimens and process them to have non-overlapping 10 partitions of 40 cm × 50 cm, as shown in Figure 7. Then, an image of each partition is divided into 80 image patches, corresponding to the actual size of 5 cm × 5 cm. Since we capture photos from two sides of two concrete specimens, the total number of image patches is 3200. We resize an image patch to 512 pixels by 512 pixels in the width and the height, and thus a pixel represents around 0.09 mm × 0.09 mm in the specimen.

In addition, domain experts in concretes materials and construction create the ground-truth of FCD manually. When generating the ground-truth, we define a crack whose width is larger than 0.3 mm or about three to four pixels as the critical crack, as recommended in [5]. The crack regions are marked with pixel intensity of 255 as the brightest pixels in an image, and vice versa for non-crack regions. It is noted that the concrete surfaces show some fire marks and paint as highlighted in the yellow boxes of Figure 7. The image patches may worsen effectiveness of the proposed network, but we include all the patches with no processing to be closer practical scenarios. We use no input normalization, noise removal, nor histogram equalization in the data preparation.

#### 4.1.2. Crack Forest Dataset (CFD) and AigleRN

The CFD has 118 RGB images with resolution of 320 × 480 captured from pavements [34]. The dataset includes sample images suffered from noise and light changes to test in various environments. AigleRN has 38 gray images with a resolution of 460 × 990 [35]. The dataset has clean crack images captured from roads, but it has complex background textures. The ground truth of the FCD is determined by domain experts [5]. The ground truth of CFD and AigleRN is available along with the datasets.

### 4.2. Training Methods

We use natural image datasets such as BSDS [37], ImageNet [38], VOC [39], DRIVE [40] in addition to the concrete crack images to avoid the overfitting problem. We show the procedures in detail as follows. 

**Figure 8:**
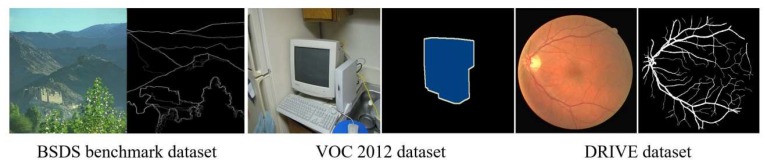
BSDS, VOC2012, and DRIVE datasets for a learning in the proposed technique.

When learning parameters of CNN from the beginning, all the parameters are initialized with random Gaussian distribution as in [41]. However, the learning method may incur an overfitting problem from an insufficient number of training samples. The collection of a large number of annotated crack images poses some challenges due to the expensive costs, e.g., in fire experiments. Therefore, we apply a learning method to use several non-crack datasets in addition to the crack datasets to efficiently learn the parameter of each sub-network. Our hypothesis is that the model parameters intensively trained on the well-annotated large-scale datasets can help the crack recognition tasks effectively. Specifically, we first use some of the convolution layers of VGG16 that have been trained using ImageNet [38] for the common layers of CRA and CCA. They are shown in white in Table 1 and Table 2. Then, we use DRIVE [40] and VOC [39] datasets to learn the layers of CRA in Table 2. The VOC and DRIVE datasets have some annotations used for image segmentation. However, we use them regardless of several types of labels. In other words, the different annotations remarking foreground regions are set to 1 while the other background regions are set to 0. Then, we train the CCA using the BSDS dataset [37]. The dataset is originally developed for image segmentation, but it remarks a ground truth of an edge in a natural image as shown in Figure 8. Thus, it can be used for training a CCA network to learn edge-like components in cracks. In this training step, the last three layers in CRA with gray colors in Table 2 are not changed. Afterwards, we use all the datasets including DRIVE, VOC, and BSDS, and FCD, CFD, and AigleRN for fine-tuning all the layers of the networks in the end-to-end learning. We use no additional optimization techniques such as batch normalization, drop-out, data augmentation, or K-fold validations in training, and therefore, the proposed learning is the only method to avoid the overfitting in our work.

### 4.3. Loss Function

The training dataset *D* = {(*X*, *Y*)} is composed of an input set of a crack image *X* and a ground truth set of a crack map *Y*. *x* ∈ *X* denotes an input sample of a crack image, and *y* ∈ *Y* denotes the ground truth. *L* is a loss function defined as a sum of three cross-entropy losses, i.e., *L*1, *L*2, and *L*3. The cross-entropy loss represents the error between the output of the cross-entropy function $\tilde{y}$ and the ground truth *y*. Mathematically, we have

(1) $$L_{i}=-\frac{1}{N}\sum_{x}\left[y\ln{\tilde{y}}_{i}+(1-y)\ln(1-{\tilde{y}}_{i})\right]$$

where ${\tilde{y}}_{1}$ and ${\tilde{y}}_{2}$ are the output images of the CCA and CRA, respectively. ${\tilde{y}}_{3}$ is the predicted output image, given as the element-wise multiplication of the CCA and CRA.

We train the network parameter $h^{*}$ to minimize *L*, i.e.,

(2) $$h^{*}=\arg\min(L)=\arg\min(\alpha_{1}L_{1}+\alpha_{2}L_{2}+\alpha_{3}L_{3}),$$

where $\alpha_{1}=\alpha_{2}=0.3$ and $\alpha_{3}=0.4$. To obtain the parameter, we use the standard backpropagation algorithm using ADAM optimizer. To be specific, the learning rate is 10^−5^, and the rate is reduced to a multiple of 0.1 every 10,000 iterations. The training is stopped after 10K iterations. The hyperparameters are empirically obtained.

## 5. Experimental Result

In this section, we evaluate the performance of the proposed algorithm. All experiments are conducted on a GPU sever with Intel 3.5GHz, 32GB memory and a GPU (Geforce GTX 1080) that is sourced from NVIDIA, Santa Clara, USA. We used Caffe deep learning software framework to implement the proposed technique. We used around 3230 training samples and 135 testing samples in FCD dataset. We combine the sample images in CFD and AigleRN datasets. The two datasets consist of sample images of pavements, while the numbers of the samples are relatively small to train the convolution neural network. We used 156 samples in the combined sets for the testing.

The detection performance of the proposed algorithm is evaluated with other conventional algorithms. Specifically, we first adopt state-of-the-art crack detection algorithms based on CNN, i.e., Cha et al. method [14] and Kim et al. method [15]. They use block-based classification techniques to identify whether or not a tested block of an image presents a crack. We also compare Lim et al. method [21], recently developed for the crack detection using the Laplacian of Gaussian (LoG). Furthermore, we employ HED network [9] and DCAN [11] for the comparisons since they are the state-of-the-arts contour and edge detection algorithms based on CNN that can be possibly applied to the crack detection.

### 5.1. Performance Comparisons for Crack Detection

**Figure 9:**
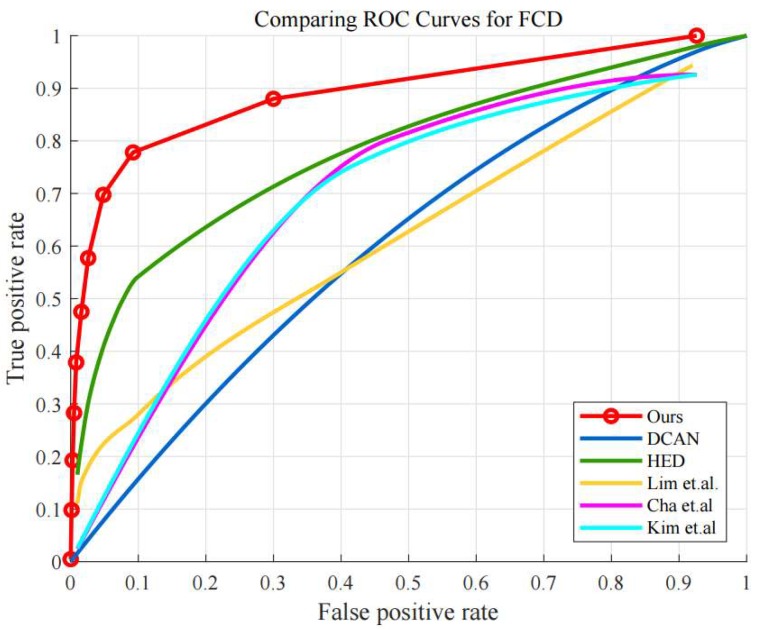
ROC curves of compared algorithms in FCD.

We examine the performance with receiver operating characteristics (ROC) curves and the corresponding area-under-curve (AUC) values. Figure 9 shows the ROC curves using FCD dataset, comparing the performance of the proposed algorithm denoted by “Ours” and the other conventional algorithms. As shown in Figure 9, the proposed algorithm outperforms all the other conventional algorithms. The AUC value of “Ours” is 0.904 while HED, DCAN, Lim et al., Cha et al., and Kim et al. are 0.779, 0.602, 0.617, 0.626, and 0.620, respectively.

**Figure 10:**
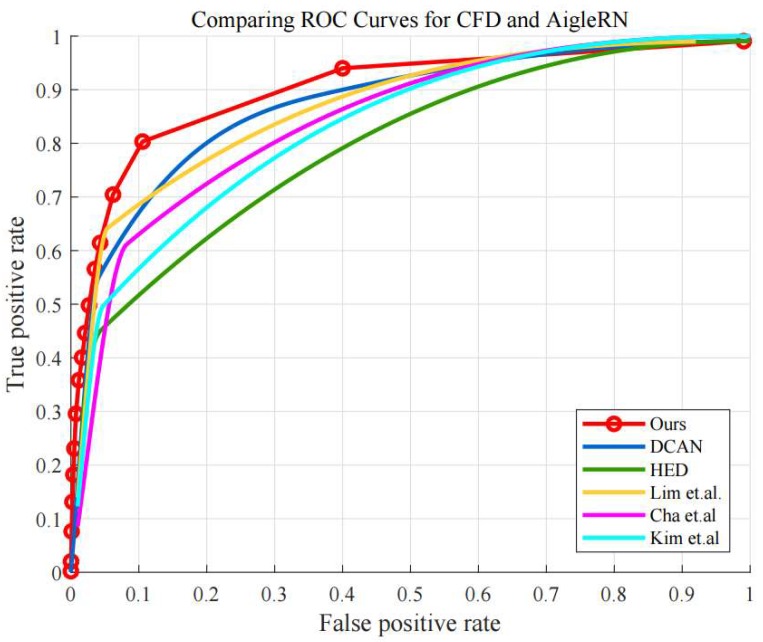
ROC curves of compared algorithms in CFD and AigleRN.

Moreover, Figure 10 shows the ROC curves using CFD and AigleRN datasets. As can be seen, the proposed algorithm also provides better detection performance than the other algorithms, while the DCAN shows comparable performance. Quantitatively, the AUC values of the proposed algorithm is 0.910, while those of the HED, DCAN, Lim et al., Cha et al., and Kim et al. are 0.795, 0.872, 0.867, 0.843, and 0.830, respectively.

It is observed that some of the compared algorithms also provide fairly good performance in the detection, when using the CFD and AigleRN datasets. One reason can be the properties of the two datasets, which contain less noise in the concrete surfaces. In comparison, the FCD dataset poses more challenges in the detection as the surfaces are sometimes contaminated by combustion, and the images display a number of small and tiny cracks, given in the fire experiments. Accordingly, most of the algorithms show worse performance. Particularly, the conventional algorithm using edge detection such as Lim’s method shows significantly different performance in Figure 9 and Figure 10. However, the performance of the proposed algorithm is relatively reliable regarding the different datasets in the quantitative results.

For more quantitative comparisons, we calculate the precision, the recall, and the F-measure. The precision is obtained with the proportion of the crack samples to the entire samples that are estimated to the crack. The recall is obtained with the proportion of the crack samples to the entire samples that actually are the crack. A $F_{\beta }$ score is the harmonic mean of the precision and the recall. It is mathematically given as

(3) $$F_{\beta }=(1+\beta^{2})\frac{precision\cdot recall}{\beta^{2}precision+recall}$$

where β is set to 1 in our evaluation. The precision, the recall, the F-measure, and the AUC values are shown in Table 3 and Table 4 when using the FCD dataset and using the CFD and AigleRN datasets, respectively. As described in the tables, the proposed algorithm yields significantly improved performance as compared to the other algorithms.

We also compare the computational complexity of the proposed algorithm with the tested algorithms in terms of the measurement time and memory sizes. We observed the time and the memory when 10 input image samples are processed and measured the numbers on the average to have robust results. In our model, the time was around 0.585 s, and the CPU and GPU memory were around 60MB and 3.8GB, respectively. As for Lim’s method, the time was around 0.512 s and the CPU and GPU memory around 42MB and 1.4GB, similar to Cha’s method. Both the methods are actually developed for block classification, and the amounts of the computational complexity were smaller than the proposed algorithm. However, the performance is somewhat degraded. As for HED and DCAN, they have deeper convolutional layers, increasing the computational loads more, and the time was estimated around 4.677 s and 1.077 s, respectively. In our design, we have attempted to use all convolution layers [42] instead of using pooling layers. The performance varies with test datasets slightly while the computational time increases. Thus, we use the pooling layers. The CPU memory were around 410MB and 380MB, and the GPU memory around 11GB and 8.2GB, which are larger than the proposed algorithm. They have deeper layers than the proposed algorithm.

### 5.2. Visual Comparisons for Crack Detection

**Figure 11:**
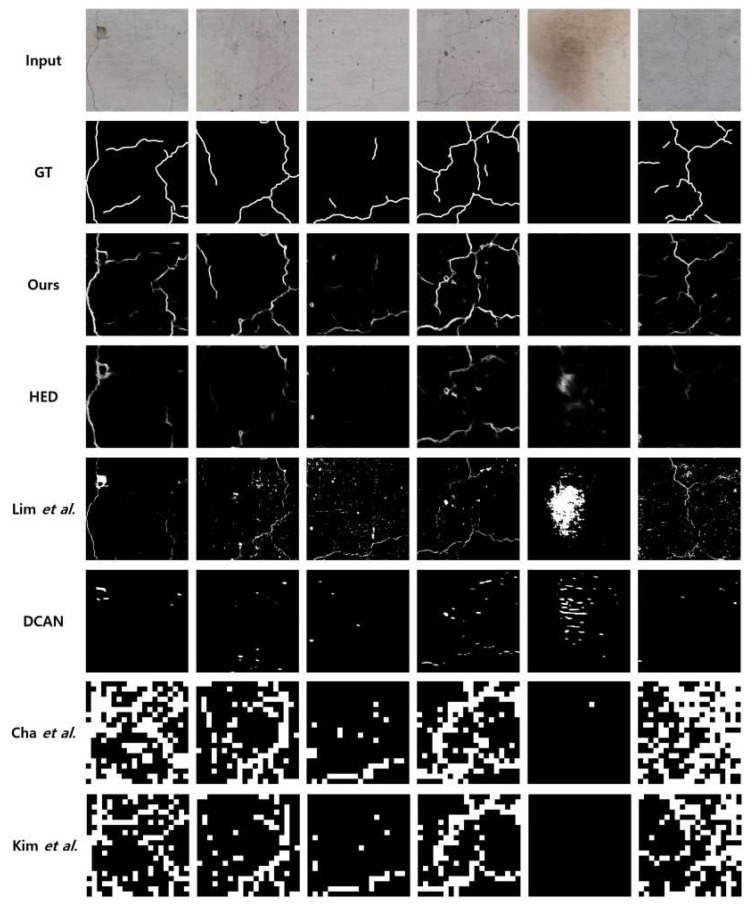
Comparisons on FCD dataset.

We perform the visual comparisons in Figure 11. A larger pixel intensity represents a higher probability of crack information in the position, determined by each algorithm. The FCD dataset has a number of image patches including stains and noise that occurred at the fire experiments, so it has challenges distinguishing the noise components and the actual cracks. As shown in Figure 11, the proposed algorithm provides better results that are closer to the ground truth, even though some cracks, e.g., in the sixth column are not detected properly. However, the other algorithms also fail to detect the cracks. Actually, the crack detection algorithms are not much efficient when the cracks are not noticeable compared to the background. As compared to Lim et al.’s method, the proposed algorithm is more robust regarding color changes. For instance, in the fifth column, when there is no crack beside stains in the image patch, the proposed algorithm outputs empty crack information. However, Lim et al.’s method outputs some amounts of false positive errors. As compared to HED and DCAN, the proposed algorithm detects the cracks better. Both Cha et al.’s method and Kim et al.’s method have similar results because they include similar classification processes done by CNN. The difference is only the use of the probability map measuring the confidence in the existence of the crack. In Kim et al.’s method, they used soft-thresholds once the block is classified. However, they predict many image patches to cracks in common, especially when the datasets contain noise. For this reason, though both of them provide higher recall values, the precision values become significantly lower, which results in significantly degraded overall accuracies in detection.

**Figure 12:**
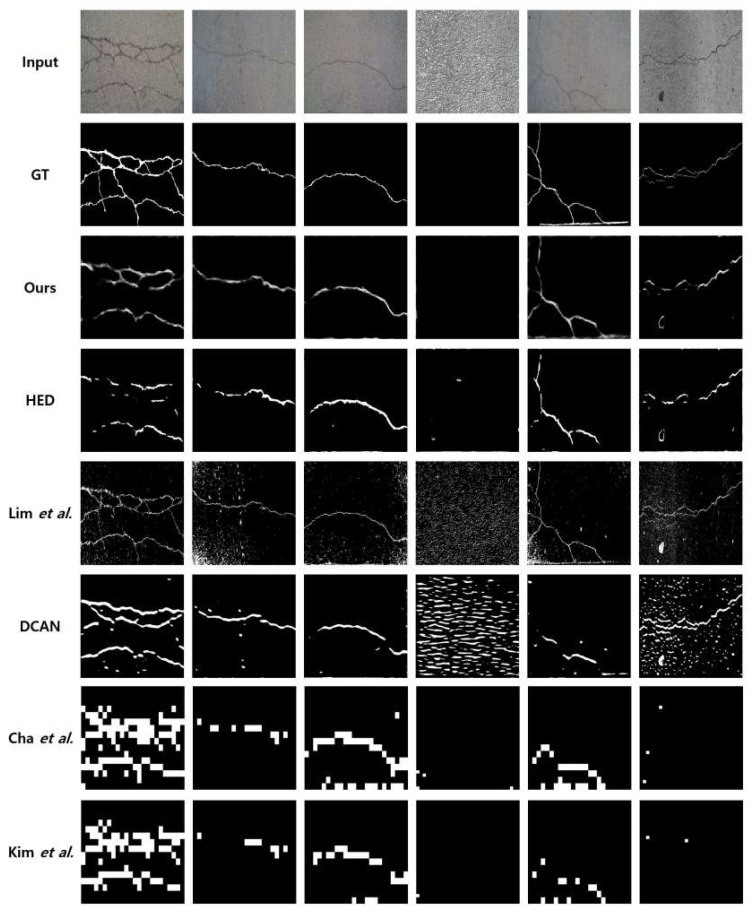
Visual comparisons on CFD and AigleRN dataset.

The visual comparisons of CFD and AigleRN are given in Figure 12. In the fourth and sixth columns, the proposed algorithm and the HED output comparable results to the ground truth. Meanwhile, Lim et al. and DCAN detect all the background textures of the crack images, which significantly drop the detection performance. Especially in the fourth column, DCAN fails to distinguish the crack and the background textures. HED successfully detects solid cracks in the third and in the fifth columns. The results are given when the backgrounds are simple. However, if the background becomes complicated as in the first column, HED has difficulties in the detection. Furthermore, Kim et al. and Cha et al. show robust performance against complex background textures and the performance is comparatively higher than that of FCD. However, the recognition rate is still low because of the lower precision values.

**Figure 13:**
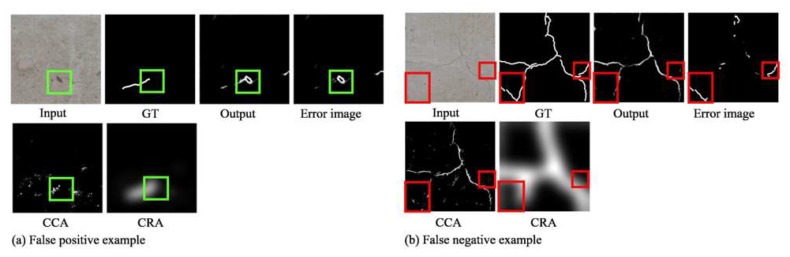
False positive and false negative example.

In Figure 13, we point out the false positive errors and the false negative errors in the proposed algorithm, by subtracting the ground truth from the output images of the proposed algorithm to obtain the error images. The green boxes represent the false positive errors, and the red boxes represent the false negative errors. For instance, in the first row of Figure 13a, the concrete image has some holes on the surface, and it is observed that the edges of the cavity are mistakenly detected as cracks. The second row of the Figure 13a is CCA and CRA output image of the first test input image. Through the CCA network, all large and small holes were detected. In the CRA, all small holes were ignored, but large holes were recognized as group of edges and detected as crack regions. As a result, large holes were detected incorrectly.

In addition, in the first row of the Figure 13b, the concrete image has both very thin and thick cracks and the thin crack is ignored when difference in thickness is large. The second row of the Figure 13b is CCA and CRA output image of that input concrete image. When there is a significant difference in thickness, the thin cracks are recognized as if the intermediate connection is broken. However, they are not clearly recognized even in the CCA network. In the CRA network, only the thick crack region was recognized, and the thin crack region was ignored. Finally, the thin cracks recognized by CCA were completely eliminated by CRA.

**Figure 14:**
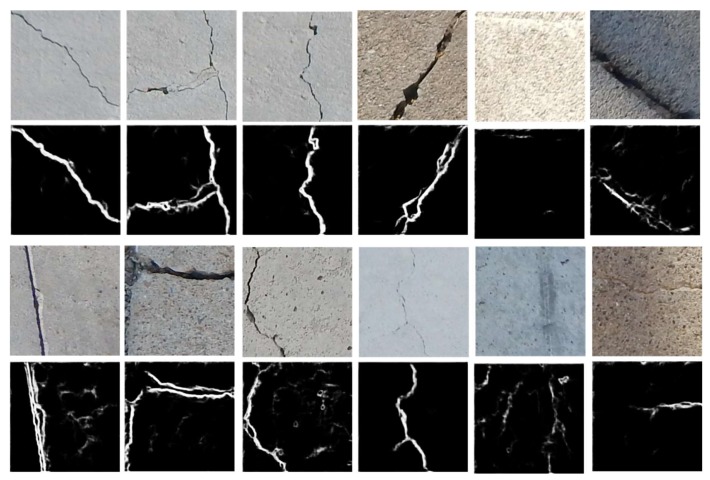
Crack detection results on SDNE dataset.

We also conduct experiments called inter-DB in Figure 14 to see the effective performance of the proposed network. The inter-DB denotes that the trained networks are applied in testing, using the different dataset that has been not used in training. The inter-DB is more challenging because the network needs to be adapted to different properties. In Figure 14, we observe the network is efficiently applied to the new dataset named SDNET2018 [43]. The last two columns of Figure 14 show interesting results. In the fifth column, there are no visible cracks in the images, but there are some color changes. The proposed network is able to capture the difference and show very few activations in the output. In the last crack, there are color changes as well as cracks. In this case, the network can capture the differences. These results show that the network can be efficiently applied in inter-DB as well.

## 6. Conclusions

We proposed an autonomous crack detection algorithm by using two-branched convolutional neural network. The cracks cannot be distinguished with other noises when they are obtained from image sensors. To efficiently recognize cracks, we designed a crack-component-aware (CCA) network with a u-shaped structure to train the edge features of the cracks. Crack-region-aware (CRA) network emphasizes critical cracks and suppresses trivial noise. Through the combination of two sub-networks, we could finally extract only the significant cracks. The proposed method requires some increments of the computational complexity due to the deep learning architectures as compared to block-based crack detection techniques. However, the proposed algorithm provided improved detection accuracies and reliable detection performance as compared to the previous algorithms in different crack image datasets.

## Figures and Tables

**Table 1:** Implementation details of the crack-component-aware network.

Layer	Kernel Size	Stride	Feature Map
Conv1_1	3	1	512 × 512 × 64
Conv1_2			
Pool1	2	2	256 × 256 × 64
Conv2_1	3	1	256 × 256 × 128
Conv2_2			
Pool2	2	2	128 × 128 × 128
Conv3_1	3	1	128 × 128 × 256
Conv3_2			
Conv3_3			
Pool3	2	2	64 × 64 × 256
Deconv1	4	2	128 × 128 × 128
Deconv2	4	2	256 × 256 × 64
Deconv3	4	2	512 × 512 × 32
1×1 Conv	1	1	512 × 512 × 1
Cross-entropy	1	1	512 × 512 × 1

**Table 2:** Implementation details of the crack-region-aware network.

Layer	Kernel Size	Stride	Feature Map
Conv1_1	3	1	512 × 512 × 64
Conv1_2			
Pool1	2	2	256 × 256 × 64
Conv2_1	3	1	256 × 256 × 128
Conv2_2			
Pool2	2	2	128 × 128 × 128
Conv3_1	3	1	128 × 128 × 256
Conv3_2			
Conv3_3			
Pool3	2	2	64 × 64 × 256
Deconv1	16	8	512 × 512 × 128
1×1 Conv	1	1	512 × 512 × 1
Cross-entropy	1	1	512 × 512 × 1

**Table 3:** Performance results on the FCD.

	Precision	Recall	F-measure	AUC
Ours	0.749	0.753	0.751	0.904
HED	0.774	0.655	0.709	0.779
DCAN	0.746	0.137	0.231	0.602
Lim et al.	0.471	0.173	0.253	0.617
Cha et al.	0.212	0.983	0.349	0.626
Kim et al.	0.169	0.833	0.281	0.620

**Table 4:** Performance results on the CFD and AigleRN.

	Precision	Recall	F-measure	AUC
Ours	0.834	0.830	0.832	0.910
HED	0.344	0.502	0.408	0.795
DCAN	0.702	0.837	0.764	0.872
Lim et al.	0.723	0.791	0.756	0.867
Cha et al.	0.266	0.935	0.414	0.843
Kim et al.	0.244	0.774	0.371	0.830
